# Agglomeration behaviour of magnetic microparticles during separation and recycling processes in mRNA purification

**DOI:** 10.1002/elsc.202000112

**Published:** 2021-06-24

**Authors:** Lars Wommer, Winda Soerjawinata, Roland Ulber, Percy Kampeis

**Affiliations:** ^1^ Environmental Campus Birkenfeld Institute for biotechnical Process Design Trier University of Applied Sciences Hoppstädten‐Weiersbach Germany; ^2^ Institute of Bioprocess Engineering Technical University Kaiserslautern Kaiserslautern Germany

**Keywords:** agglomeration, de‐agglomeration, high‐gradient magnetic separation, magnetic beads, mRNA‐vaccines

## Abstract

Purification of mRNA with oligo(dT)‐functionalized magnetic particles involves a series of magnetic separations for buffer exchange and washing. Magnetic particles interact and agglomerate with each other when a magnetic field is applied, which can result in a decreased total surface area and thus a decreased yield of mRNA. In addition, agglomeration may also be caused by mRNA loading on the magnetic particles. Therefore, it is of interest how the individual steps of magnetic separation and subsequent redispersion in the buffers used affect the particle size distribution. The lysis/binding buffer is the most important buffer for the separation of mRNA from the multicomponent suspension of cell lysate. Therefore, monodisperse magnetic particles loaded with mRNA were dispersed in the lysis/binding buffer and in the reference system deionized water, and the particle size distributions were measured. A concentration‐dependent agglomeration tendency was observed in deionized water. In contrast, no significant agglomeration was detected in the lysis/binding buffer. With regard to magnetic particle recycling, the influence of different storage and drying processes on particle size distribution was investigated. Agglomeration occurred in all process alternatives. For de‐agglomeration, ultrasonic treatment was examined. It represents a suitable method for reproducible restoration of the original particle size distribution.

**Abbreviations**
(m)RNA: polyadenylated ribonucleic acid
DNA: desoxy ribonucleic acid
dT: desoxythymidine
HGMS: high‐gradient magnetic separation
LiDS: lithium dodecyl sulfate
mRNA: messenger ribonucleic acid
OD_600_
: optical density at λ = 600 nm
Tris(‐HCl): Tris‐(hydroxymethyl)‐aminomethan(‐hydrochloride)
TT buffer: Tris Tween buffer
USP: United States Pharmacopeia

## 1 INTRODUCTION

### 1.1 Magnetic separation in mRNA vaccine production

High‐gradient magnetic separation (HGMS) can be used to selectively separate magnetizable components from suspensions. This technique has already been applied by various working groups in the field of biotechnology as well [1, 2, 3, 4, 5]. It was utilized for the separation of immobilized enzymes [6, 7, 8] and used for the isolation of target molecules [9, 10, 11, 12, 13, 14, 15]. In this process, the target molecule is specifically adsorbed on the functionalized particle surface in a reaction mixture and desorbed again from the magnetic particles (called magnetic beads) after magnetic separation has taken place. An overview of widespread magnetic separators and magnetic particle systems is given in [16, 17]. One widely used microparticle system in the bioseparation of proteins, mRNA, and viruses is Dynabeads [18].

Currently of increasing importance in the field of biopharmacy is the production of mRNA for vaccine manufacturing [19, 20, 21]. The synthesis of a mRNA vaccine is described in the literature [19, 22–27]. Within the production of a vaccine, separation and purification of mRNA are necessary in the process. This could be done via affinity chromatography using deoxythymidine oligo(dT) cellulose or by use of polystyrene latex particles to which oligo(dT) functionalization is attached [28]. Also, mRNA can be purified with oligo(dT) magnetic particles, with a sequence of 14‐25 thymine bases [29]. For this purpose, established, automatable laboratory protocols already exist on the mL scale [17, 30, 31]. In these protocols, several buffer changes and washing steps are performed, involving successive magnetic separation and redispersion procedures. The cell suspension used to prepare the mRNA is usually washed and centrifuged to obtain a cell pellet. Lysis/binding buffer is then added to the cell pellet to initiate lysis of the cells, which is carried out by a repeated passage of the solution through a pipette tip. Annealing of the mRNA produced with the cells to the functionalized magnetic particles also takes place in the lysis/binding buffer at room temperature with shaking. After magnetic separation, the mRNA‐loaded oligo(dT)_25_ magnetic particles are first washed with two different wash buffers to remove non‐specific adhering impurities. Before eluting the mRNA, the last wash buffer must be removed and Tris‐HCl buffer is added to the particles. Elution of the mRNA is performed at elevated temperature. The supernatant contains the mRNA and could be clarified by a further magnetic separation.

**PRACTICAL APPLICATION**

Magnetic microparticles have gained importance in the purification of mRNA‐based vaccines. They serve as adsorbents for the mRNA. Through several steps of magnetic separation followed by redispersion of the magnetic beads for washing and elution, the mRNA can be isolated. This is usually done on a mL scale. To obtain larger amounts of mRNA, flow‐through magnetic separation using high‐gradient magnetic separation (HGMS) can be advantageous. Here, the suspension to be processed is then usually in a stirred feed tank. Due to particle‐particle interactions, a not inconsiderable agglomeration may occur, especially due to the attached mRNA. This can reduce the mRNA yield. Furthermore, for economic reasons it is necessary to perform magnetic particle recycling, for which there are various process alternatives. With regard to a possible use of HGMS in an mRNA production for vaccines, particle size distributions were determined to investigate the agglomerating or de‐agglomerating effect of different process steps.

By transferring these laboratory protocols 1:1 to small‐scale production using HGMS, no process steps of an already established cell culture‐based process would have to be changed. Thus, a time saving in the production of required mRNA quantities for clinical studies could be realized. To verify the up‐scalability of mRNA purification after cell lysis from millilitre to litre scale with HGMS according to existing laboratory protocols, experiments were performed, in which magnetic beads suspensions were stirred in a feed vessel. Since the lysis/binding buffer usually contains a detergent and a high salt concentration compared to the other buffers involved, it was used as the most relevant buffer system of cell culture‐based mRNA purification (see Sections 2.2.1 and 3.1.2). Due to the basic research, no real cell lysate was used for the experiments. The loading step with synthetic mRNA was performed with already purified polyadenylated RNA.

### 1.2 Impact of agglomeration in bioseparation with magnetic particles

Magnetic particles interact and agglomerate with each other when a magnetic field is applied [32, 33]. It is further known that mRNA aggregates due to interactions of complementary base pairs and their sequences, respectively [34, 35, 36]. Cross‐linking of RNA molecules via proteins can also take place [37]. Therefore, it is expected that particle agglomeration may also occur during the above described magnetic separation steps due to mRNA loading. Agglomeration of the magnetic particles is undesirable because the agglomeration of the magnetic beads reduces the total functional surface area, which may result in yield losses during hybridization and elution of mRNA. Mandel et al. [38] described this reduced surface area causing longer diffusion pathways that decrease adsorption speed for molecules. Also, Hoffmann et al. [13] concluded to avoid agglomeration because of reduced yields in adsorption and elution of molecules. The aggregation of magnetic particles often reduces the functional surface to such an extent, that the loading of the target molecule decreases drastically [39]. Hubbuch et al. [10] emphasized the need for avoiding agglomeration of the particles at all costs in the field of protein adsorption and elution. The elution yield of, for example, green fluorescent protein from agglomerated particles was very low compared to a suspension with non‐agglomerated particles [40].

The particle size distribution can therefore be regarded as a characteristic parameter to indirectly describe the expected loss of mRNA hybridization and elution yield. When magnetic beads agglomerate, their total surface area containing the functional oligo(dT) is reduced due to the interparticle contact areas. Because of their non‐porous character, the reduced functional outer‐surface cannot be neglected. A positive aspect of agglomeration, however, is that it leads to improved separation in the magnetic separation process [33, 41–44]. In order to be able to make statements about the agglomeration behaviour of oligo(dT)_25_‐functionalized and mRNA‐loaded magnetic particles, particle size distributions were determined.

Magnetic particle systems are high‐priced products that have to be recovered in a technical process from an economic point of view. Agglomeration is very likely to occur during the (intermediate) storage to be provided in this process. It might even make sense to include a drying step in the magnetic particle recycling. Usually, toxic preservatives like natrium azide up to concentrations of 0.2% are added to storage buffers of magnetic beads to prevent spoilage of microorganisms [30, 45]. The particles have to be washed intensively prior to re‐use to remove traces of the preservatives. For application in vaccine purification, even low concentrations of these substances have to be avoided in any case. An alternative storage method for the particles is dry storage, which does not require any preservatives. During resuspension, agglomeration must also be expected. To evaluate the agglomerating effect of these process steps, particle size measurements were carried out after storage or drying/resupending of the magnetic particles. In addition, the effect of ultrasonic treatment on de‐agglomeration was investigated.

## 2 MATERIALS AND METHODS

### 2.1 Preparation of mRNA‐loaded oligo(dT)_25_ magnetic particles

#### 2.1.1 Coupling Dynabeads MyOne Carboxylic Acid with oligo(dT)_25_

Dynabeads MyOne Carboxylic Acid (Life Technologies AS, Oslo) are uniformly spherical and monodisperse magnetic particles with a diameter of 1.05 μm ± 0.03 μm [18] and functionalization with carboxyl groups. Their physical and chemical parameters are given in [18, 33, 46, 47]. The content of carboxyl groups is 0.6 mmol·g^–1^ [47]. Due to the higher content of functional groups per gram of beads, these particles are favourable with respect to a high yield of mRNA compared to 2.8 μm Dynabeads M‐270 Carboxylic Acid. However, Dynabeads MyOne in oligo(dT)‐functionalized form are not commercially available and were prepared as part of this work. For this purpose, Dynabeads MyOne Carboxylic Acid were coupled with oligo(dT)_25_.

Amino‐functionalized oligonucleotides were purchased from Invitrogen Life Technologies AS. Between the amino group at the 5′ end, six carbon atoms were attached as spacers, followed by 25 bases of thymine in the 3′ direction. The molecular weight was 7724 g·mol^–1^. The particle concentration was 10 mg·mL^–1^ according to the manufacturer, of which 0.5 mL was taken after shaking for 30 min. The tube was then placed in the stand magnet for 2 min and the supernatant was withdrawn. The Dynabeads MyOne Carboxylic Acid were washed two times in 1 mL of MES buffer (Applichem, ≥99%) and a volume of 50 μL was adjusted in MES buffer. To perform the coupling, an EDC‐MES solution was prepared from 240 mg *N*‐ethyl‐*N*'‐(3‐dimethylaminopropyl)carbodiimide hydrochloride (EDC, Merck) in 1 mL of 100 mM MES buffer with pH = 4.8, which was freshly prepared before each coupling.

A volume of 30 μL of the amino‐oligo(dT)_25_ solution with a concentration of 0.83 nmol·μL^–1^ was mixed with 20 μL of the EDC‐MES buffer solution. Then, the carboxylated Dynabeads in MES buffer were combined with this amino‐oligo(dT) preparation and vortexed for 10 s, after which the particle concentration was 50 mg·mL^–1^. The suspension was then incubated for 16 h at room temperature in an overhead shaker. The particles were then incubated three times with TT buffer for 30 min. A concentration of oligo(dT)_25_ magnetic particles of 5 mg·mL^–1^ in PBS buffer was then adjusted. This protocol was used to verify the coupling process of amino‐oligo(dT)_25_ to Dynabeads MyOne Carboxylic Acid and scaled up for the coupling of 1.75 g magnetic particles.

Sarkar et al. [48] describe a coupling of amino‐functionalized oligo(dT)_25_ to carboxylated magnetic particles in a sonicator. It was observed that the oligo(dT)_25_ covalently bound to the magnetic beads withstood the harsh conditions and was not transferred into solution [49].

#### 2.1.2 Preparation of polyadenylated RNA

In cells, the proportion of mRNA is only 1‐5% of the cells’ total RNA of 10‐30 pg [30, 48, 50]. Therefore, the amount of mRNA that can be isolated directly from cells might be too small for hybridization of magnetic particles to detect the effect of mRNA loading on agglomeration behaviour. To overcome this problem, polyadenylated RNA – as a “synthetic mRNA”, so to speak – was prepared as follows in order to be able to present a large input quantity. This synthetic mRNA will be referred to as (m)RNA in the following.

*Escherichia coli* BW3110 with plasmid pJOE 4056.2_6His_eGFP [51] from a 300 μL glycerol cryoculture was grown in a shake flask with 150 mL LB medium containing 150 μL 10% ampicillin and incubated at 37°C and 120 rpm for approximately 16 h. All subsequent steps took place in accordance with the respective kit instructions. The *GeneJET Plasmid Miniprep* kit (ThermoFisher) was used for plasmid DNA isolation. Restriction digestion of the plasmid was performed using the HindIII‐HF restriction enzyme (New England Biolabs). The enzyme was then inactivated at 80°C for 20 min at 300 rpm in a thermal shaker (HLC MKR 13, Ditabis AG). The DNA was then in linearized form and purified using the *DNA Clean & Concentrator‐5 (Capped)* kit (Zymo Research).

This linearized plasmid DNA was concentrated using a rotary vacuum concentrator (RVC2‐18, Martin Christ Gefriertrocknungsanlagen GmbH) for 30 min untempered at 1500 rpm and 610 Pa. By the *AmpliScribe T7 Flash Transcription* kit (Lucigen Corporation) the DNA was translated into complementary RNA with a length of about 900 bases. Purification of RNA was performed via precipitation with 3.854 g ammonium acetate (Roth, ≥97%) dissolved in 10 mL ultrapure water. The purified RNA was then polyadenylated using the *A‐Plus Poly(A) Polymerase Tailing* (Cellscript) kit. An UV/Vis spectrophotometer (DS‐11, DeNovix Inc.) was used to determine the concentration of DNA and polyadenylated RNA.

#### 2.1.3 Hybridization of polyadenylated RNA to oligo(dT)_25_‐functionalized Dynabeads

Hybridization of polyadenylated RNA was performed according to the prescription of the *mRNA purification* kit of commercially available 2.8 μm oligo(dT)_25_‐Dynabeads, used to separate mRNA from total RNA [30]. Therefore, an initial (m)RNA concentration of 666.7 μg·mL^–1^ in water and a magnetic bead concentration of 5 mg·mL^–1^ were chosen. After washing the magnetic beads with 0.1 mL wash buffer and 0.1 mL binding buffer, another 0.1 mL binding buffer was added and mixed with 0.1 mL (m)RNA. The hybridization of (m)RNA to oligo(dT)_25_‐functionalized Dynabeads was carried out by shaking at room temperature for 5 min. Due to the synthetic nature of the (m)RNA, a binding buffer [30] was utilized for pre‐loading the particles instead of the lysis/binding buffer used in mRNA purification from cell cultures (see Section 2.2.1). After washing two times with 0.2 mL wash buffer (see Section 2.2.2), elution took place by adding 20 μL elution buffer, heating up to 80°C for 2 min and removing the supernatant. This protocol was used for verification and scaled up to hybridize the needed amount for loading of 1.75 g of available oligo(dT)_25_‐Dynabeads MyOne. Hybridization was detected in the supernatant after elution via UV/VIS measurement using the UV/Vis spectrophotometer (DS‐11, DeNovix Inc.).

### 2.2 Buffer systems

#### 2.2.1 Buffers in mRNA purification

##### Lysis/binding buffer in mRNA purification

The lysis/binding buffer consisted of 15.76 g of Tris‐HCl (Applichem, ≥99%), 21.20 g of LiCl (Roth, pure ≥98.5%) and 10.0 g of SDS (Roth, ultrapure) in 1 L ultrapure water. The pH of 7.5 was adjusted with 1 M NaOH (Roth, 1 N measured solution) [52]. Wang et al. and also Petersen et al. used an identical lysis buffer with the exception that they used LiDS instead of SDS [53, 54]. Note: Storage experiments in lysis/binding buffer of up to 3 days took place (see Section 3.2). Therefore, the substances DTT and EDTA, which are often used in lysis buffers, have been omitted, as their use in conjunction with Dynabeads is not recommended [55]. However, these substances were used in the other buffers because they are present there in lower concentrations and the contact time to the Dynabeads is short.

##### Wash buffers A and B during mRNA purification

The wash buffer A consisted of 1.576 g Tris‐HCl (Applichem, ≥99%), 6.359 g LiCl (Roth, pure ≥98.5%), 0.372 g EDTA‐Na_2_ * 2H_2_O (VWR, ≥99%), and 1.0 g SDS (Roth, ultrapure) in 1 L ultrapure water. pH was adjusted to 7.5 with 1 M NaOH (Roth, 1 N measured solution) [29, 52, 54]. Wash buffer B consisted of the same components as wash buffer A, except that it did not contain SDS or LiDS [52, 53, 54].

##### Elution buffer in mRNA purification

The elution buffer consisted of 1.576 g Tris‐HCl (Applichem, ≥99%) in 1 L ultrapure water. pH was adjusted to 7.5 with 1 M NaOH (Roth, 1 N measured solution) [29, 52, 54].

#### 2.2.2 Buffers in hybridization of polyadenylated RNA

##### MES buffer in hybridization of polyadenylated RNA

The MES buffer consisted of 2.13 g MES (Merck) in 0.1 L ultrapure water. pH was adjusted to 4.8 with 1 M NaOH (Roth, 1 N measured solution) [56].

##### Tris buffer in hybridization of polyadenylated RNA

The 1 M Tris buffer consisted of 30.3 g Tris (Roth, ≥99.9%) in 0.25 L ultrapure water. pH was adjusted to 8.0 with 37% HCl (AnalaR NORMAPUR) [56].

##### TT buffer in hybridization of polyadenylated RNA

The TT buffer consisted of 250 mL of 1 M Tris buffer with pH = 8.0 and 1 mL of 10% Tween 20 solution (Merck) in 1 L ultrapure water [56].

##### Binding buffer in the hybridization of polyadenylated RNA

The binding buffer consisted of 0.242 g Tris (Roth, ≥99.9%), 4.239 g LiCl (Roth, pure ≥98.5%) and 0.074 g EDTA‐Na_2_ * 2H_2_O (VWR, ≥99%) in 0.1 L ultrapure water. pH was adjusted to 7.5 with 37% HCl (AnalaR NORMAPUR) [30].

##### Wash buffer in the hybridization of polyadenylated RNA

The wash buffer consisted of 0.121 g Tris (Roth, ≥99.9%), 0.636 g LiCl (Roth, pure ≥98.5%) and 0.037 g EDTA‐Na_2_ • 2H_2_O (VWR, ≥99%) in 0.1 L ultrapure water. pH was adjusted to 7.5 with 37% HCl (AnalaR NORMAPUR) [30].

##### Elution buffer in hybridization of polyadenylated RNA

The elution buffer consisted of 0.121 g Tris (Roth, ≥99.9%) in 0.1 L ultrapure water. pH was adjusted to 7.5 with 37% HCl (AnalaR NORMAPUR) [30].

##### PBS buffer in hybridization of polyadenylated RNA

The PBS buffer consisted of 0.294 g NaH_2_PO_4_ • 2H_2_O (Merck, ≥99%), 1.44 g Na_2_HPO_4_ • 2H_2_O (Roth, ≥98%), and 8.78 g NaCl (AnalaR NORMAPUR, >99.5%) in 1 L ultrapure water [47].

### 2.3 Drying techniques for oligo(dT)_25_‐Dynabeads MyOne

For the drying of oligo(dT)_25_‐Dynabeads MyOne suspensions, different drying techniques were applied. As a first alternative, drying was performed in a drying oven at 70°C (T6 Heraeus Oven, Thermo Fisher Scientific Inc.). Sample volumes of 1‐5 mL each were dried until constant weight was achieved. As a second alternative, drying with a rotary vacuum concentrator (RVC2‐18, Martin Christ Gefriertrocknungsanlagen GmbH) was tested. For this purpose, sample volumes of 0.5‐1 mL each were processed untempered at T = 26‐41°C for a period of 3‐5 h at 1500 rpm and 610 Pa. Freeze‐drying was used as a third alternative. For this purpose, sample volumes of 1 mL particle suspensions were frozen overnight at ‐20°C. Freeze‐drying was performed at 360 Pa in a freeze‐dryer (Alpha 1–2 LDplus, Martin Christ Gefriertrocknungsanlagen GmbH).

### 2.4 High‐gradient magnetic separation

High‐gradient magnetic separation was performed with a magnetic separator (HGF‐10, Steinert Elektromagnetebau GmbH) and a self‐developed 3D‐printed separator chamber as described in [57]. The suspension to be processed with volumes of V = 2.15 L or V = 2.7 L was stirred in a 3 L measuring beaker made of styrene‐acrylonitrile copolymer (VITLAB GmbH). Therein, stirring was carried out with a four‐bladed propeller stirrer with a diameter of d = 10 cm (R1345, IKA‐Werke GmbH & Co. KG) at a speed of 220 rpm.

### 2.5 Measurement of particle concentration by means of turbidity measurement

An UV/Vis spectrometer (Genesis 10, Thermo Scientific) at a wavelength of 600 nm was used for turbidity measurements of particle suspensions. Measurements were made in 10 × 4 × 45 mm half‐micro polystyrene cuvettes (Sarstedt AG & Co. KG). Turbidity measurements were performed using a 1:40 dilution with deionized water or the buffer system used. The (magnetic) filtrate resulting from the HGMS was measured undiluted.

### 2.6 Determination of the particle size distribution

#### 2.6.1 Laser diffraction

The determination of the particle size distribution was performed as described in [57]. Therefore, a laser particle sizer with small‐volume liquid dispersion unit (Analysette 22 MicroTec, Fritsch GmbH) was used with the associated software *MaS control V1.00.009*. In the software, Fraunhofer theory with the setting “very narrow” was chosen because of its very small TradeOff parameter and Root Mean Square error. Thereby, the measured values are displayed correctly, without any additional smoothing of the results like in other settings. From the cumulative distribution, the median values of the volume distribution d_3,10_, d_3,50_, und d_3,90_ were determined in the software, of which the d_3,50_ value was used to characterize the particle size distribution.

#### 2.6.2 Brightfield microscopy

A cell counter (CellDrop BF, DeNovix Inc.) was used for qualitative evaluation of particle size within suspensions due to the small sample volume and the fast and simple measurement. A sample volume of 10 μL was always pipetted into the counting chamber. The focus remained set at 710 for each measurement. The instrument is actually recommended for counting cells of a size between 4 and 400 μm. However, since on the one hand the tests carried out here concerned 1 μm particles and on the other hand even agglomerates were not counted correctly, the measurement result was not suitable for quantification. Nevertheless, it was used to optically evaluate the particle size measurements as well as the influences of particle treatment steps.

### 2.7 Ultrasonic homogenization

An ultrasonic sonotrode (consisting of GM 2200, HD 2200, KE 76, Bandelin electronic GmbH & Co. KG) with an operating frequency of 20 kHz and a power of 200 W was used to disperse or de‐agglomerate magnetic particle suspensions. The sonotrode tip was immersed 3 cm into the sample, which was thereby placed in a 50 mL Falcon tube or a 1 L measuring cup, respectively and sonicated at an amplitude of 19‐23%.

### 2.8 Agglomeration experiments in the feed vessel after ultrasonic homogenization

For agglomeration experiments without performing HGMS, (m)RNA‐preloaded magnetic beads were used (see Section 2.1.3) and suspended in deionized water or lysis/binding buffer. So, no real cell lysis was performed, even in case of using lysis/binding buffer. Particle suspensions with a volume of 0.4 L were placed in a 1 L measuring cup made of styrene‐acrylonitrile copolymer (VITLAB GmbH) as a feed vessel. After ultrasonic homogenization (see Section 2.7), a four‐bladed propeller stirrer d = 5 cm (R1342, IKA‐Werke GmbH & Co. KG) was operated with a stirrer drive (RW 16 basic, IKA‐Werke GmbH & Co. KG) at a speed of 500 rpm. The ratio of stirrer diameter to feed vessel diameter was d/D = 0.556. Subsequently, sampling was performed at defined intervals of 5 min. All agglomeration experiments took place at a temperature of 20°C in a water bath (MC‐E, Peter Huber Kältemaschinenbau).

### 2.9 Effects of ultrasonic treatment on the bonding of functional groups and on (m)RNA re‐loading

Coupling of Dynabeads MyOne Carboxylic Acid with oligo(dT)_25_ was performed according to Section 2.1.1. Then, six samples of 1 mg of the oligo(dT)_25_‐Dynabeads MyOne were washed two times with 0.5 mL deionized water including vortexing. Note: Due to the adsorption of particles on the plastic surface especially after magnetic separation when MES buffer was used, complete resuspension was not possible. Thereby, non‐reacted amino‐oligo(dT)_25_ could be entrapped by the particle agglomerates analogously as mentioned in Section 1.2. After washing, those 6 mg magnetic beads were transferred to a 50 mL Falcon tube and filled up to a volume of 15 mL and ultrasonication was performed (see Section 2.7). The supernatant of this suspension was concentrated via rotation vacuum concentration (see Section 2.3) until a volume of 40 μL was obtained. To detect the possible loss of covalently bound oligo(dT)_25_ from Dynabeads MyOne, UV/VIS measurements of the concentrated supernatant were carried out using an UV/Vis spectrophotometer (DS‐11, DeNovix Inc.). To evaluate the influence of the ultrasonic treatment on the (m)RNA re‐loading, two samples of 1 mg of these magnetic beads were re‐loaded with (m)RNA according to Section 2.1.3. Comparisons were made to two samples of 1 mg of non‐ultrasonic treated oligo(dT)_25_‐Dynabeads MyOne.

## 3 RESULTS AND DISCUSSION

### 3.1 Behaviour of magnetic particles in a production process

#### 3.1.1 Observed agglomeration of magnetic particles in the feed vessel during magnetic separation

If the magnetic particle‐based separation technique is to be carried out in a technical scale production of mRNA‐based vaccine, it is not possible by using the magnetic separators used in the laboratory scale. Magnetic separators suitable for this purpose based on the principle of high‐gradient magnetic separation (HGMS), which are operated in through‐flow (“magnetic filters”), have been and are being developed in particular by Franzreb [1, 2, 58, 59] and in own work [6–8, 57, 60]. The magnetic particle suspension to be processed — in this case oligo(dT)_25_‐functionalized magnetic beads or mRNA‐loaded oligo(dT)_25_‐functionalized magnetic beads — is then placed in a feed vessel, which is stirred to avoid segregation due to the gravitational field. In addition, cell disruption takes place there in the lysis/binding buffer. In contrast to cell disruption on a laboratory scale, the stirrer also contributes significantly to cell disruption. It is known that intensive stirring causes high shear stress, which damages the cells [61, 62]. Nienow et al. [63] describe a stirred‐tank reactor in a suitable scale for the production of biological products for clinical trials. The lysis is initiated by adding the lysis buffer and supported by increasing the stirrer speed afterwards. Transferred to the application of oligo(dT)‐magnetic beads, a lysis/binding buffer can be added instead of the lysis buffer. After hybridization of mRNA to the magnetic beads, separation via HGMS could take place.

For HGMS of magnetic particle suspensions with volumes >2 L, a 3D‐printed separation chamber was used (see Section 2.4). It consists of a very dense transverse rhombic filter matrix arrangement for the separation and recycling of 1 μm magnetic particles. Due to the special design, the filter matrix itself is enclosed by the plastic material (with USP Class VI certification) and thus has no contact with the mRNA‐loaded particles [57].

The experiments performed here were designed to investigate, whether the loading of mRNA on the one hand and the interaction with the buffer on the other hand has a noticeable effect on the agglomeration behaviour and thus on the separation performance of the magnetic separation. For this purpose, oligo(dT)‐functionalized magnetic beads were used (see Section 2.1.1). The yield of oligo(dT) coupling was 37.87%, resulting in an oligo(dT)‐functionalization of the particles of 13.01 nmol·mg^–1^. A polyadenylated RNA, which served as a “synthetic mRNA” and will be referred to as (m)RNA hereafter, was prepared as described in Section 2.1.2. The (m)RNA loading of the particles after hybridization (see Section 2.1.3) was 8.464 μg·mg^–1^ with an (m)RNA‐hybridization yield of 12.70%. To examine the influence of this (m)RNA loading on separation performance, two magnetic separations were performed in deionized water at the same conditions. One magnetic separation was conducted with Dynabeads MyOne Carboxylic Acid and compared to the magnetic separation of oligo(dT)_25_‐Dynabeads MyOne loaded with (m)RNA. The separation chamber was characterized in [57]. The volumetric flow rates in the HGMS experiments were 100 mL·min^–1^.

In the first experiment, a suspension of 1.390 ± 0.032 g Dynabeads MyOne Carboxylic Acid was filled up with 0.4 L deionized water and treated with ultrasonication according to Section 2.7. Afterwards, the volume was adjusted to 2.15 L in the feed vessel of the magnetic separation to get a magnetic beads concentration of c_B_ = 0.647 g·L^–1^. The 1:10 diluted suspension had an OD_600_(feed) of 0.671 at a median particle diameter of d_3,50_ = 1.104 μm. In the second experiment, (m)RNA‐loaded oligo(dT)_25_‐Dynabeads MyOne were used. After ultrasonication of 0.4 L suspension with c_B_ = 4.367 ± 0.061 g·L^–1^ according to Section 2.7, the volume was adjusted to 2.70 L (c_B_ = 0.647 ± 0.009 g·L^–1^) and processed. This resulted in a turbidity of OD_600_(feed) = 0.494 of the 1:10 diluted sample with a median particle diameter of d_3,50_ = 1.944 μm.

**FIGURE 1:**
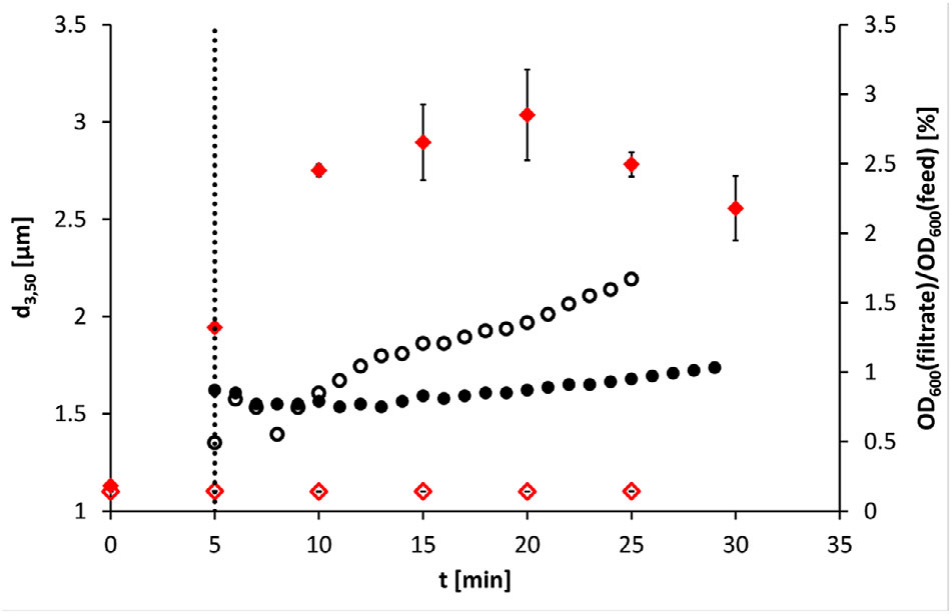
Time course of the turbidity in the (magnetic) filtrate during HGMS of (m)RNA‐loaded oligo(dT)_25_‐functionalized Dynabeads MyOne (0.65 g·L^–1^) at a flow rate of 100 mL·min^–1^ with a self‐developed 3D‐printed separation chamber normalized to the turbidity of the input suspension (●); median particle size d_3,50_ in the feed vessel during magnetic separation (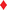); hollow symbols correspond to the same experimental set‐up with Dynabeads MyOne Carboxylic Acid without any further functionalization (error bars can be seen in the symbols); start of the HGMS feed pump marked with a vertical dotted line

Samples were taken in both experiments from the (magnetic) filtrates every minute and the OD_600_ was measured undiluted. Figure 1 shows the time course of the turbidity measurements in the filtrates normalized to the turbidities of the feed suspensions. Usually, such time course of the magnetic particle concentration in the (magnetic) filtrate (breakthrough curve) is used to evaluate the separation efficiency of the magnetic separator. Observing the curve of (m)RNA‐loaded particles (see Figure 1) shows that the turbidity in the filtrate decreased after 7 min and then remained almost constant over approximately 8 min. Thereafter, the turbidity increased steadily. This effect may have been caused either by the magnetic separator itself, or by a change in particle size in the feed vessel. In case of the unfunctionalized magnetic beads, the time course of the turbidity in the magnetic filtrate was quite similar but with a higher slope. Since there was no increase in the mean particle size, the retention of the magnetic particles was more difficult here [57].

During the magnetic separation, the median particle diameter in the feed vessel was additionally measured every 5 min to determine possible changes in the particle size distribution. The median particle diameter with Dynabeads MyOne Carboxylic Acid was in the range of d_3,50_ = 1.101‐1.105 μm. No agglomeration was observed in this experiment (see Figure 1). In the case of (m)RNA‐loaded oligo(dT)_25_‐functionalized Dynabeads MyOne, it was found that the mean particle diameter in the feed vessel had increased by 0.808 μm to d_3,50_ = 2.752 μm already 5 min after the start of the feed pump of HGMS. Subsequently, only minor changes were observed. This could be due to the fact that volume reduction at constant agitator speed leads to increased power input per volume and thus to increased shear forces. Therefore, the number of agglomerates formed could decrease from 20 min onwards, or agglomerates already formed could be broken up again.

The observed minimum in the turbidity of the filtrate corresponds approximately to the maximum in the median particle diameter in the feed vessel (see Figure 1). Therefore, the increasing turbidity is caused either by the upcoming breakthrough of the magnetic particles or by the decreasing median particle diameter. Thus, statements about the separation efficiency of the magnetic separator used, are questionable. Comparing both experiments, it can be summarized that the agglomeration was caused by the (m)RNA‐loading of the magnetic beads. The particle agglomerates supported the magnetic separation and resulted in a higher separation efficiency.

#### 3.1.2 Investigations on the agglomeration behaviour in the feed vessel

**FIGURE 2:**
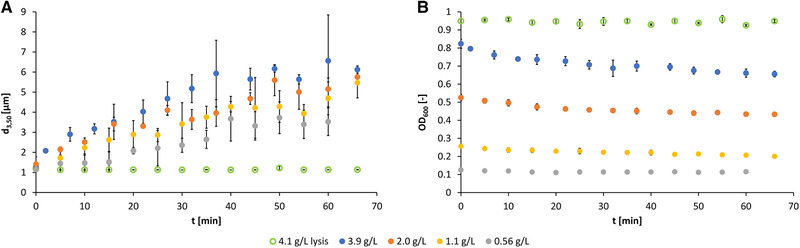
Time course of the median particle diameter d_3,50_ of (m)RNA‐loaded oligo(dT)_25_‐functionalized Dynabeads MyOne in the feed vessel at different particle concentrations in deionized water and in the lysis/binding buffer (A); course of the OD_600_ values in the corresponding experiments (B), error bars are partially visible in the symbols

Based on the observation of agglomeration of (m)RNA‐loaded particles (see Section 3.1.1), further investigations were carried out on the agglomeration behaviour in the feed vessel as described in Section 2.8 without performing HGMS. Due to the problems described in Section 1.1, the investigations were to be carried out not only in deionized water but also in a process‐relevant buffer system. In the individual magnetic separation steps in the process shown above, four relevant buffers can be identified (see Section 2.2.1). If these buffers are compared, it is noticeable that they differ only slightly in terms of substances. In particular, the lysis/binding buffer could most likely show effects on the particle size distribution due to the high concentration of buffer components compared to the other buffers. In this buffer, hybridization of mRNA from a multicomponent suspension of the cell lysate occurs. Accordingly, this is the most critical process step for the design of the magnetic separation. Therefore, the experiments presented below focus on the lysis/binding buffer. As a reference system, deionized water was used. Figure 2 shows the course of the median particle diameter (A) and the turbidity (OD_600_) in the feed vessel (B) plotted over the experimental run time.

Since all experiments were to be performed as triplicate measurements, the original particle size distribution had to be restored during the repetition. This was achieved by sonicating the entire volume of 400 mL for 1 min using an ultrasonic sonotrode with a power input of 52.3 kW⋅m^–3^, which will be further described in detail in Section 3.3.

At all prepared concentration levels in deionized water, there was clearly recognizable agglomeration, expressed both in increased d_3,50_ values and correspondingly in decreasing OD_600_ values. The influence of increasing mean particle diameters on decreasing OD_600_ values was described in [57]. As mentioned in Section 1.2, the interactions between the particles due to (m)RNA loading were responsible for this. An increase in particle size occurred especially in the first 5 min. The higher the particle concentration was, the faster agglomerates were formed. This is due to the increased contact probabilities of the magnetic particles. It can be stated that the (m)RNA‐loaded magnetic beads in the feed vessel agglomerate in deionized water, even when the suspension is stirred relatively intensively.

In comparison, the highest possible concentration of 4.1 g·L^–1^ with the available mass of 1.65 g of (m)RNA‐loaded Dynabeads was adjusted in lysis/binding buffer to investigate the agglomeration tendency in this buffer (see Figure 2). There was no significant increase in d_3,50_ values due to surfactant SDS and the high salt concentrations in this buffer, which can prevent agglomeration [44, 60]. SDS is an anionic detergent used to denature proteins in the cell lysate, but it can also attach to the magnetic beads. Due to the resulting surface charge, it could have a beneficial effect on dispersion stability.

Agglomeration effects are usually increased with a higher amount of magnetic beads [32, 64]. If no agglomeration tendency can be observed at this concentration, it would not occur at lower concentrations. Thus, agglomeration in the lysis/binding buffer does not pose a problem, i.e. there is no need to worry about yield losses of mRNA due to agglomeration here. However, this only applies if a sufficiently high energy input during hybridization in the lysis/binding buffer is ensured by a stirring system, since the magnetic particles are fed with a highly concentrated suspension to the feed vessel. It should be noted, however, that the lower the concentration of the buffer components, the more agglomeration can occur, as shown by the reference system water.

However, the non‐agglomeration in the lysis/binding buffer places high demands on the separation performance of the magnetic separator, because small particle sizes must be reliably separated.

### 3.2 Influence of drying processes on particle size distribution

**FIGURE 3:**
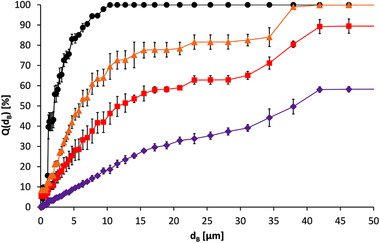
Particle size sum curves of oligo(dT)_25_‐functionalized Dynabeads MyOne; initial suspension (●) and after different drying procedures followed by resuspension in deionized water, oven drying (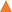), rotary vacuum concentration (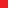) and freeze drying (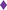)

In order to investigate the effects of different types of drying on the particle size distribution, a suspension (c_B_ = 4.833 g⋅L^–1^) was first treated for three times each 1 min with an ultrasonic sonotrode with a power input of 55.7 kW⋅m^–3^. This resulted in a median particle diameter of d_3,50_ = 2.034 ± 0.053 μm. Samples were then taken for oven drying, rotary vacuum concentration, and freeze drying. After the respective drying, the particles were resuspended in deionized water for the particle size measurements. From Figure 3, it can be seen that each drying method contributed to the agglomeration of the particles. Thereby, the particle size distribution increased significantly from oven drying to rotary vacuum concentration to freeze drying.

The median particle diameters were d_3,50_ = 6.210 ± 0.612 μm after drying in the drying oven, d_3,50_ = 12.069 ± 1.157 μm after rotary vacuum concentration and d_3,50_ = 38.194 ± 0.968 μm after freeze drying, respectively. Thus, all drying processes have a negative influence on the particle size distribution.

**FIGURE 4:**
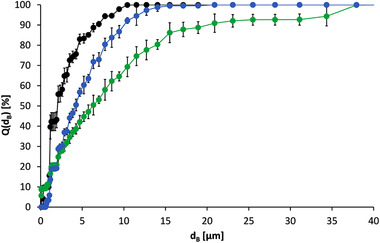
Particle size sum curves of oligo(dT)_25_‐functionalized Dynabeads MyOne; before storage (●), after storage at 6°C for 16 h (●) and in after a freeze/thaw process (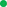)

Based on this result, storage of the aqueous suspension at reduced temperature (6°C) and in a frozen state were therefore additionally considered as further process alternatives in the recycling process. However, both methods also lead to significant agglomerate formation (see Figure 4). The median particle diameter increased from d_3,50_ = 2.034 ± 0.053 μm to d_3,50_ = 4.232 ± 0.199 μm when the aqueous suspension was stored for 16 h at 6°C. In the case of a freeze/thaw process the same initial median particle diameter increased to d_3,50_ = 5.99 ± 0.263 μm. The agglomerating effect of these two storage methods was thus in the range of drying in the drying oven.

Furthermore, storage in lysis/binding buffer at reduced temperature was also investigated. Overnight storage at 6°C (approx. 15 h) in lysis/binding buffer led to median particle diameters between d_3,50_ = 1.446 ± 0.404 μm and d_3,50_ = 3.107 ± 1.069 μm by stirring only (without ultrasonic treatment). When the storage time at 6°C was increased to 3 days, the median particle diameter was d_3,50_ = 5.062‐10.338 μm. The freezing of magnetic particles in lysis/binding buffer was also tested. Here, a magnetic particle suspension (c_B_ = 4.791 g·L^–1^) was frozen at ‐20°C and subsequently thawed. The median particle diameter before freezing was d_3,50_ = 1.167 μm. After thawing, the value increased to d_3,50_ = 2.941 ± 0.179 μm. Agglomeration was thus lower in both cases in comparison to that in deionized water, but it still occurred.

It remains to be noted that agglomeration must always be assumed in the case of magnetic particle recycling together with the storage times that occur in the process. Since this reduces the total functional surface area and can therefore lead to yield losses during hybridization or elution of mRNA, de‐agglomeration must always be carried out prior to reuse. Therefore, a suitable process step for de‐agglomeration must be included in an mRNA production process that intends to use functionalized magnetic beads in a recirculation process.

### 3.3 Influence of ultrasonic redispersion on particle size distribution

Since any drying process and even overnight storage at reduced temperature (refrigerator) leads to particle agglomeration (see Section 3.2), the establishment of a de‐agglomeration technique is required. Therefore, the suitability of the ultrasonic sonotrode, which has already been used for de‐agglomeration by Shaikh [60], was investigated in more detail.

The power input of the ultrasonic sonotrode into the respective suspension was determined following Rotoarinoro et al. [65] according to the principle of calorimetric power determination. For this purpose, 0.4 L of deionized water was poured into a 1 L measuring cup or 15 mL into a 50 mL Falcon tube, respectively. Both were tempered in a water bath (MC‐E, Peter Huber Kältemaschinenbau) at 20°C for 1 h before sonication. After sonication, the temperature was measured with a thermometer (Checktemp 1, Hanna Instruments). Sonification took place for 1 min in 0.4 L and 10 s in 15 mL, respectively, both at 19‐23% power. Power consumption was calculated using the specific heat capacity of water at 20°C (4.183 kJ⋅kg^–1^⋅K^–1^) according to Equation 1.

(1) $$\frac{P}{V}=\frac{\rho \cdot c_{p}\cdot \Delta T}{\Delta t}$$

The ultrasonic treatment resulted in power inputs of 52.3 kW⋅m^–3^ for 0.4 L and 1352 kW⋅m^–3^ for 15 mL.

#### 3.3.1 Application of ultrasound to aqueous stored magnetic particle suspensions

**FIGURE 5:**
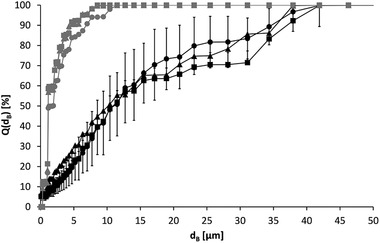
De‐agglomeration of (m)RNA‐loaded oligo(dT)_25_‐functionalized Dynabeads MyOne (4 g·L^–1^) by 1‐minute ultrasound treatment with a power input of 52.3 kW⋅m^–3^; suspensions before ultrasound: 1●, 2▲ (single measurement), 3■; suspensions after ultrasound 1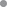, 2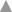, 3
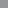

**FIGURE 6:**
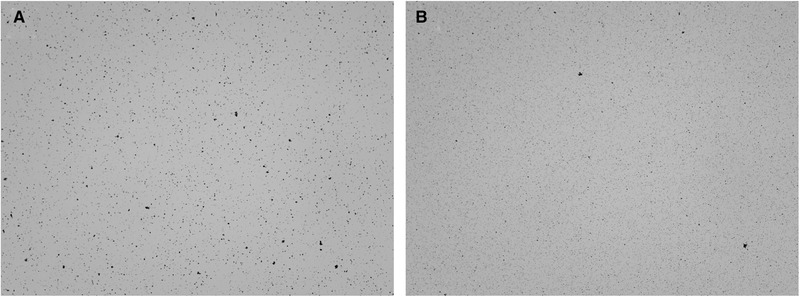
Microscopic images of suspensions (dilution 1:40, sample volume 10 μL) from the feed vessel containing (m)RNA‐loaded oligo(dT)_25_‐functionalized Dynabeads MyOne (3.3 g·L^–1^) in deionized water after storage at 6°C for 15 h and stirring for 1 h (A) and after subsequent 1‐minute ultrasound treatment with power input of 52.3 kW·m^–3^ in 400 mL volume (B)

For de‐agglomeration, sonication intervals of 1 min with a power input of 52.3 kW⋅m^–3^ were applied. With this, it was possible to achieve the desired de‐agglomeration in a volume of 0.4 L (see Figure 5). Experiments 1 and 2 in Figure 5 were carried out immediately after each other. Therefore, stirring was performed for about 160 min before experiment 2, so that the agglomeration at the beginning of experiment 2 was close to that of experiment 1. Between experiments 2 and 3, storage in the refrigerator overnight was carried out and agglomeration occurred (see Section 3.2). The initial suspensions had median particle diameters of d_3,50_ = 11.418 μm ± 3.141 μm; 10.150 μm and 11.354 ± 1.937 μm. Due to the experimental procedure, only single determinations of particle size distribution can be made after ultrasonic treatment. Median particle diameters of d_3,50_ = 1.597 μm, d_3,50_ = 1.151 μm, and d_3,50_ = 1.151 μm, could be determined. Thus, de‐agglomeration occurred as desired in all cases. For qualitative evaluation, multiple determinations from 1:40 diluted samples were made using a cell counter. The observations revealed that isolated agglomerates in the range of 3 to 10 μm were still present (see Figure 6B). Overall, however, there was a qualitatively good and largely homogeneous de‐agglomeration (see difference between Figure 6A and Figure 6B).

The ultrasonic treatment presented above thus provides a method for reproducible and nearly complete de‐agglomeration of (m)RNA‐loaded oligo(dT)_25_‐functionalized Dynabeads. This result is in accordance with the work of Shaikh [60], who obtained with a different particle system containing 3‐5 μm magnetic particles (Carboxylated magnetic particles M‐PVA C22, PerkinElmer chemagen Technologie GmbH).

The influence of ultrasonic treatment on oligo(dT)_25_‐functionalization was investigated according to Section 2.9. After sonification, the concentrated supernatant showed UV/Vis absorption corresponding to an oligo(dT)_25_ loss of 8.57%. This means a loss of 1.115 nmol·mg^–1^ of functional groups per bead mass. It is even possible, that washing of non‐reacted amino‐oligo(dT)_25_ by means of vortexing was not sufficient (see Section 2.9). In this case, the ultrasound treatment dispersed the particles so well, that only now these unbound biolinkers could be released. Therefore, the values given above may be too high. Also, (m)RNA‐loading capacity after ultrasonication was investigated in binding buffer as described in Section 2.9. In contrast to the agglomeration in MES buffer, the magnetic beads were well resuspended in binding buffer. Therefore, falsification by agglomeration such as in the MES buffer (see above) is not likely here. After de‐agglomeration, with a power input of 52.3 kW·m^–3^, no loss of (m)RNA loading capacity was observed. This means that the apparent loss of oligo(dT)_25_ indicated above must result mainly from unbound residues between the particle agglomerates. Nevertheless, regardless of the results presented here, it must be investigated whether ultrasonic treatment (possibly with higher power input) leads to destruction or detachment of the surface functionalization. Compromises may have to be found in the relationship between the resulting particle size and consistent functionality.

#### 3.3.2 Application of ultrasound to dried and redispersed magnetic particles

**FIGURE 7:**
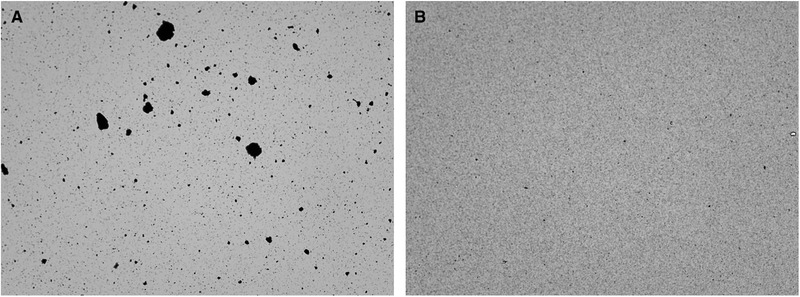
Microscopic images of suspensions (undiluted) with oligo(dT)_25_‐functionalized Dynabeads MyOne (1 g·L^–1^) after freeze‐drying and resuspension in deionized water (A) and after subsequent 10 s ultrasonic treatment with power input of 1352 kW⋅m^–3^ in 15 mL volume (B)

In Section 3.2, it was found that freeze‐drying of magnetic particle suspensions results in significant agglomeration of the particles. The process is the least suitable drying process in terms of agglomerating effect and thus places the highest demands on de‐agglomeration. Therefore, de‐agglomeration with the ultrasonic sonotrode was examined for freeze‐dried and resuspended particles. Sonication of 15 mL suspension took place at a specific power input of 1352 kW⋅m^–3^ in a 50 mL Falcon tube. Due to the smaller volume that could be sonicated here, the sonication time was reduced to 10 s. A further theoretically possible reduction of the sonication time due to the volume reduction from 400 to 15 mL could not be realized with the available device. The median particle diameter was successfully reduced from d_3,50_ = 29.297 ± 5.55 μm to d_3,50_ = 1.109 ± 0.001 μm. Figure 7 shows images of the cell counter before and after ultrasound treatment. It can be seen, that de‐agglomeration occurred approximately completely here as well.

## 4 CONCLUDING REMARKS

When deionized water was used to prepare magnetic particle suspension from oligo(dT)_25_‐functionalized Dynabeads loaded with (m)RNA, a pronounced agglomeration behaviour was observed in the feed vessel, due to the (m)RNA loading. Agglomeration increased with increased particle concentrations due to the greater contact probability of the particles. Agglomeration occurred mainly in the first 5 min of the experiment. Therefore, even with the usual resuspension time of 1–3 min for magnetic particles, a significant increase in particle size can be assumed. In lysis/binding buffer, on the other hand, no increase in particle size of stirred suspensions could be detected ‐ presumably due to the dispersion‐stabilizing effect of the SDS contained therein.

However, this means that when designing magnetic separators, care must be taken to ensure that the magnetic separator is capable of quantitatively retaining magnetic particles with small average particle diameters. When optimizing HGMS chambers for mRNA‐based vaccine production, suspensions containing lysis/binding buffer should preferably be used. In the case of other buffers, it is important to ensure that the original particle size distribution in the feed vessel is maintained during the experiments, e.g. by repeated ultrasound treatment.

Some drying and storage methods were investigated for their potential use in a recycling process. Drying or storage resulted in increased mean particle diameters after redispersion for each method. Therefore, a process step for de‐agglomeration must be included in the process in any case. Irrespective of the investigations carried out here, it is of course still necessary to check how drying affects the functionality of the particles. Ultrasonic treatment using a sonotrode was able to restore the original particle size distribution of oligo(dT)_25_‐functionalized Dynabeads in suspensions both after storage in aqueous media and after application of various drying processes with subsequent resuspension. Table 1 summarizes the influences of different storage conditions, drying methods and ultrasonic treatment on the mean particle diameter of oligo(dT)_25_‐Dynabeads MyOne suspensions.

**Nomenclature**
c: [g⋅L^‐1^] Concentration
c_p_
: [kJ⋅kg^‐1^⋅K^‐1^] Specific heat capacity
d: [m] Diameter (of the stirrer or particle)
D: [m] Diameter of the vessel
d_3,50_
: [μm] Median particle diameter of the volume distribution
OD_600_
: [‐] Turbidity at λ = 600 nm
P: [kW] Power
t: [s] Time
T: [°C] Temperature
V: [L] Volume

**Greek symbols**
ρ: [kg⋅m^‐3^] Density
λ: [nm] Wavelength

**Indices**
B: magnetic beads

**TABLE 1:** Influence of the process steps on the mean particle diameter of oligo(dT)_25_‐Dynabeads MyOne

Particle treatment step	d_3,50_ before treatment [μm]	d_3,50_ after treatment [μm]	c_B_ [g·L^‐1^]
(m)RNA‐loaded oligo(dT)_25_‐Dynabeads MyOne			
Stirring in the feed vessel with water	1.300 ± 0.0210	6.122 ± 0.183	3.867 ± 0.450
Stirring in the feed vessel with lysis/binding buffer	1.196 ± 0.0693	1.136 ± 0.009	4.133 ± 0.047
Ultrasonic in the feed vessel with water	10.974 ± 0.583	1.300 ± 0.210	3.867 ± 0.450
Ultrasonic in the feed vessel with lysis/binding buffer	Not necessary		
Recycling of oligo(dT)_25_‐Dynabeads MyOne			
Storage in water 16 h	2.034 ± 0.053	4.232 ± 0.199	4.833 ± 0.094
Storage in lysis/binding buffer 15 h	1.136 ± 0.009	1.45–3.11	4.133 ± 0.047
Storage in lysis/binding buffer 3 d	1.15	5.06–10.34	4.133 ± 0.047
Freezing/thawing in water	2.034 ± 0.053	5.99 ± 0.263	4.833 ± 0.094
Freezing/thawing in lysis/binding buffer	1.17	2.941 ± 0.179	4.791 ± 0.107
Oven drying	2.034 ± 0.053	6.210 ± 0.612	4.833 ± 0.094
Rotation vacuum concentration	2.034 ± 0.053	12.069 ± 1.157	4.833 ± 0.094
Freeze drying	2.034 ± 0.053	38.194 ± 0.968	4.833 ± 0.094
Ultrasonic after freeze drying/resuspension	29.297 ± 5.55	1.109 ± 0.001	4.100 ± 0.163

## CONFLICT OF INTEREST

The authors have declared no conflicts of interest.

## Data Availability

The data that support the findings of this study are available from the corresponding author upon reasonable request.
